# Photoelectrochemical sensor based on Au/Fe_3_O_4_ for ultrasensitive detection of uric acid corresponding to alzheimer’s disease

**DOI:** 10.3389/fchem.2026.1782669

**Published:** 2026-02-13

**Authors:** Xin Wang, Bin Wu, Jian An, Yan Cai

**Affiliations:** 1 Department of Neurology, Taihe County People’s Hospital, Taihe, China; 2 School of Chemistry and Chemical Engineering, Nantong University, Nantong, China

**Keywords:** Alzheimer, Au/Fe3O4, photoelectrochemical sensor, ultrasensitivity, uric acid

## Abstract

**Introduction:**

Uric acid (UA) is a crucial biochemical indicator in the human body. The dynamic balance between its production and excretion is essential for maintaining homeostasis, and detecting UA concentration enables disease diagnosis. To address the limitations of traditional UA detection methods, such as high cost and complex operation, this study constructed a photoelectrochemical (PEC) sensor modified with Au/Fe_3_O_4_.

**Methods:**

Fe_3_O_4_ and Au nanoparticles (Au NPs) were synthesized via hydrothermal methods, and the Au/Fe_3_O_4_ composite was prepared by ultrasonically loading Au NPs onto the surface of Fe_3_O_4_.

**Results:**

Under visible light illumination, the Au/Fe_3_O_4_ composite exhibited a significant photocurrent response to UA, primarily due to the synergistic effect between the localized surface plasmon resonance (LSPR) induced by Au NPs and the photogenerated electron–hole pairs from Fe_3_O_4_. This synergy promotes the redox reaction of UA at the electrode surface, thereby enhancing the photocurrent signal. Under optimized conditions, the Au/Fe_3_O_4_/GCE showed a good linear relationship in the range of 0–100 μmol/L with a detection limit as low as 3.3 μmol/L (S/N = 3).

**Discussion:**

The sensor demonstrated excellent anti-interference ability and stability, offering a new approach for UA detection. This method holds promise for practical applications in clinical diagnosis and bioanalysis.

## 1 Introduction

Uric acid (UA) is the final product of purine metabolism in humans, primarily produced in the liver and excreted by the kidneys. UA plays complex physiological and pathological roles and is a vital biomarker in clinical medicine and disease diagnosis (Selvam et al., 2020). And physiologically, UA is a key antioxidant in the body. The dynamic balance between its production and excretion is crucial for maintaining health. Under normal conditions, serum UA concentration remains within a stable range. Elevated serum UA levels can lead to metabolic syndrome and hyperuricemia, which may further cause complications such as gouty arthritis, hypertension, and coronary heart disease (Copur et al., 2022). Conversely, low serum UA levels may be associated with Parkinson’s disease or multiple sclerosis (Gagliardi et al., 2009; Lippi et al., 2008; Lohsoonthorn and Williams, 2006). Therefore, with the increasing number of patients affected by diseases related to abnormal UA levels, there is a need for rapid, efficient, stable, and highly sensitive methods for UA detection.

Common UA detection methods include enzymatic analysis, high-performance liquid chromatography, spectrophotometry, and electrochemical assays (Sun et al., 2016; Wang et al., 2015; Zuo et al., 2008). Although these methods offer high accuracy, they generally suffer from limitations such as high equipment costs, complex procedures, and long analysis times, making them unsuitable for point-of-care testing. To overcome these limitations, researchers have turned to novel sensing technologies. Advances in nanomaterials and biosensing have led to the development of new detection platforms such as electrochemical sensors, photoelectrochemical (PEC) devices, and fluorescent biosensors (Wang et al., 2025a). These technologies, with their advantages of fast response, high sensitivity, and operational simplicity, have become a focus in UA detection research. In recent years, PEC sensors have emerged as a promising approach due to their high sensitivity, excellent selectivity, stability, and practicality. The photoelectric material is a key factor influencing the performance of PEC sensors.

Gold nanoparticles (Au NPs), typically 1–100 nm in size, possess unique physical and chemical properties. They are easily functionalized and their surfaces can be modified with various functional molecules (e.g., thiol compounds, biomolecules) via chemical bonds, enabling specific detection of target analytes (Tao et al., 2024). Additionally, Au NPs exhibit excellent biocompatibility, catalytic activity, and electrical properties, showing great potential in biomedical, catalytic, and sensing applications. Fe_3_O_4_ is an excellent photoactive material that, under illumination, generates photogenerated electrons and holes, which participate in redox reactions at the electrode surface to produce a photocurrent signal for target detection (Wang Li. et al., 2020b; Zhang et al., 2025). Furthermore, the difference between the bandgap of Fe_3_O_4_ with other composite materials can facilitate more efficient carrier separation and transfer. Due to its active surface and ease of functionalization, Fe_3_O_4_ nanomaterials can be surface-functionalized (e.g., with antibodies/aptamers, immobilized enzymes) to construct highly specific bio-recognition interfaces. Combined with photoelectric signal amplification, this enables trace detection of targets such as tumor markers, nucleic acids, and small-molecule metabolites (with detection limits down to nM or fM levels) (Xiang et al., 2026). The magnetic separation capability of Fe_3_O_4_ significantly improves the pretreatment efficiency of complex biological samples (e.g., serum, cell lysates) while reducing matrix interference, offering an efficient technical approach for point-of-care testing (POCT) (Hofer, 2021; Van et al., 2025). Fe_3_O_4_, as a magnetic material, facilitates electrode recovery and reuse, while Au enhances light absorption and electron transfer efficiency, potentially improving the sensitivity and selectivity of UA detection (Chen et al., 2021).

**SCHEME 1:**
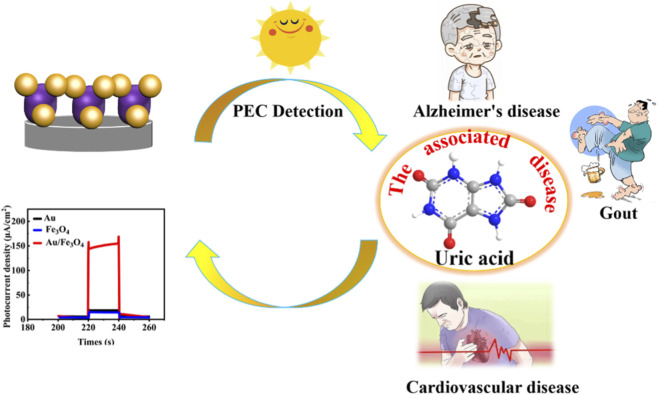
Schematic representation of the Au/Fe_3_O_4_ PEC sensing process of uric acid.

In this study, Au NPs and Fe_3_O_4_ were synthesized via hydrothermal methods, and the Au/Fe_3_O_4_ nanocomposite prepared by ultrasonic compounding was used for highly sensitive UA detection. Fe_3_O_4_ possesses unique magnetic properties, good biocompatibility, and catalytic performance, while Au exhibits excellent LSPR effects and conductivity. Their combination in a PEC sensor achieves complementary advantages and synergistic enhancement, as illustrated in Scheme 1. The successfully fabricated Au/Fe_3_O_4_ PEC sensor enables rapid, highly sensitive, and selective UA detection. This method is expected to facilitate clinical UA detection and provide theoretical support for understanding the relationship between Alzheimer’s disease and uric acid concentration.

## 2 Experimental section

### 2.1 Preparation of Au, Fe_3_O_4_, and Au/Fe_3_O_4_

#### 2.1.1 Synthesis of Au nanoparticles

Au NPs were synthesized via a hydrothermal method (Zhang et al., 2023). Firstly, 0.9 mL citric acid and 2.1 mL 0.1 mol/L sodium citrate were rapidly added to 150 mL of boiling distilled water and stirred for 15 min. Then, 1 mL 25.4 mmol/L chloroauric acid was added to the mixture, followed by stirring for an additional 3 min. The reaction solution was immediately quenched in an ice-water bath. The product was centrifuged, washed, and the obtained precipitate was dispersed in 10 mL of distilled water for subsequent use.

#### 2.1.2 Synthesis of Fe_3_O_4_ nanoparticles

A mixture of 0.675 g FeCl_3_·6H_2_O, 1.8 g CH_3_COONa, and 0.5 g polyethylene glycol was dispersed in a certain amount of ethylene glycol solvent. After thorough stirring, the mixture was continuously stirred at 50 °C for 2 h until it turned brown. The solution was then transferred to an autoclave and heated at 200 °C for 4 h. After reaction, the product was washed three times with distilled water and ethanol to remove impurities. After centrifugation, the obtained Fe_3_O_4_ nanospheres were left to stand for 48 h, then fully cooled and dried for later use.

#### 2.1.3 Preparation of Au/Fe_3_O_4_ nanocomposite

A certain amount of Fe_3_O_4_ nanoparticles was dispersed in ethanol and sonicated for 30 min to form a uniform suspension. This suspension was transferred to a container containing the Au NPs solution and stirred under ultrasonication for 1.5 h to allow thorough compounding. Finally, the Au/Fe_3_O_4_ composite was separated using a magnet, washed repeatedly with ethanol and deionized water to remove unreacted substances and impurities, and dried in a vacuum oven to obtain the Au/Fe_3_O_4_ composite.

#### 2.1.4 Fabrication of Au/Fe_3_O_4_/GCE

A glassy carbon electrode (GCE) was first polished with alumina powders of different particle sizes (500 nm and 50 nm). To further clean the electrode, the polished GCE was ultrasonicated in absolute ethanol and ultrapure water for 30 s each. Then, 10 μL Au/Fe_3_O_4_ composite dispersion was drop-coated onto the clean, dry electrode surface and dried under an infrared lamp at 50 °C, resulting in the Au/Fe_3_O_4_/GCE.

## 3 Results and discussion

### 3.1 Characterization of Au, Fe_3_O_4_, and Au/Fe_3_O_4_

**FIGURE 1:**
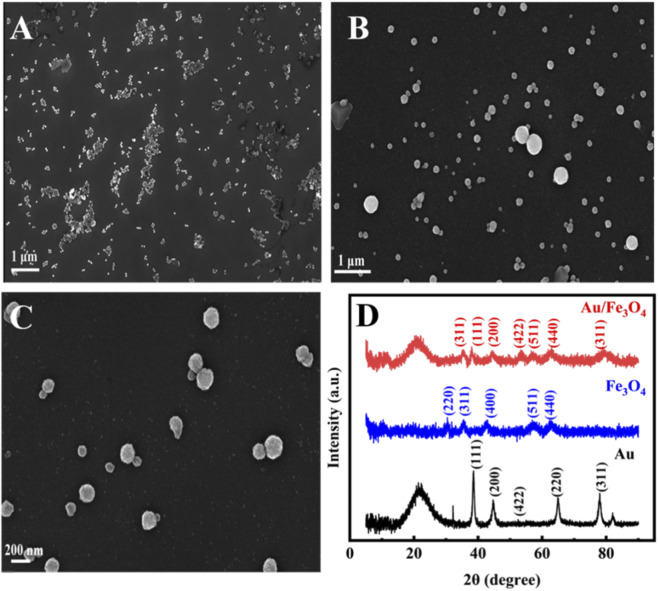
SEM images of Au **(A)**; Au/Fe_3_O_4_ **(B) (C)** and XRD pattern of Au/Fe_3_O_4_ **(D)**.

Figures 1A–C shows the scanning electron microscopy (SEM) images of Au NPs, Fe_3_O_4_, and Au/Fe_3_O_4_, respectively. The Au NPs exhibited uniform spherical or quasi-spherical morphology (Figure 1A). The Fe_3_O_4_ nanoparticles appeared spherical with a size of approximately 200 nm (Figure 1B). In the Au/Fe_3_O_4_ composite (Figure 1C), Au NPs were dispersed on the surface of the Fe_3_O_4_ nanospheres. X-ray diffraction (XRD) was used to investigate the crystal structure (Figure 1D). The characteristic diffraction peaks of Au NPs appeared at 2θ = 38.21°, 43.96°, 53.45°, 62.69°, and 75.95°, corresponding to the (111), (200), (422), (220), and (311) crystal planes, respectively (Bhardwaj et al., 2024). For Fe_3_O_4_, the characteristic peaks were observed at 30.66°, 35.42°, 43.02°, 57.09°, and 62.69°, corresponding to the (220), (311), (400), (511), and (440) planes, respectively (Cheng et al., 2017). The XRD pattern of Au/Fe_3_O_4_ (red curve) showed peaks at 35.42°, 38.21°, 43.96°, 53.45°, and 62.69°, corresponding to the (311) plane of Fe_3_O_4_ and the (111), (200), (422), and (220) planes of Au, confirming the successful synthesis of the Au/Fe_3_O_4_ nanocomposite (Wang et al., 2025b).

### 3.2 Electrochemical and photoelectrochemical performance of Au, Fe_3_O_4_, and Au/Fe_3_O_4_

**FIGURE 2:**
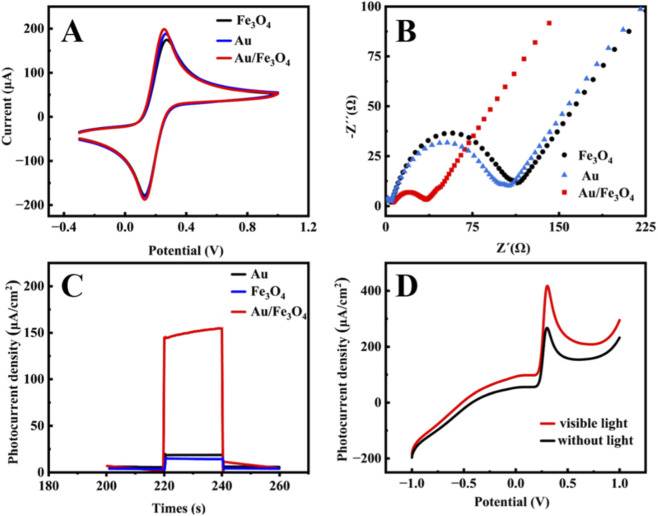
**(A)** Cyclic voltammetry curves and **(B)** electrochemical impedance spectroscopy of Au, Fe_3_O_4_ and Au/Fe_3_O_4_ electrodes in 5 mM K_3_ [Fe(CN)_6_]/K_4_ [Fe(CN)_6_] and 0.1 M KCl solution. **(C)** Transient photocurrent and time curves in PBS (pH = 7.0) containing 50 μM uric acid. The light source was xenon lamp, interrupted every 20 s. **(D)** Differential pulse voltammetry curves of Au/Fe_3_O_4_ in PBS (pH = 7.0) containing 50 μM uric acid in the presence and absence of light.

Figure 2A presents the cyclic voltammetry (CV) curves of Au, Fe_3_O_4_, and Au/Fe_3_O_4_ modified electrodes in a solution containing 5 mM K_3_ [Fe(CN)_6_]/K_4_ [Fe(CN)_6_] and 0.1 M KCl. The Au/Fe_3_O_4_-modified electrode showed a higher oxidation peak current compared to electrodes modified with Au or Fe_3_O_4_ alone. This is attributed to the good redox capability of Fe_3_O_4_ and the excellent charge transfer ability of Au NPs, whose LSPR effect accelerates charge transfer and suppresses electron–hole recombination (Jian et al., 2021). Electrochemical impedance spectroscopy (EIS) was used to evaluate the conductivity (Figure 2B). The semicircle diameter followed the order: Au/Fe_3_O_4_ < Au < Fe_3_O_4_, indicating that Au/Fe_3_O_4_ exhibited the best charge transfer ability, consistent with the CV results (Inico et al., 2025). Figure 2C displays the transient photocurrent–time response curves in PBS (pH 7.0) containing 50 μM UA under visible light illumination (xenon lamp, interrupted every 20 s). The photocurrent density increased in the order: Au/Fe_3_O_4_ > Au > Fe_3_O_4_, with the composite showing the highest response. Figure 2D compares the differential pulse voltammetry (DPV) responses of the Au/Fe_3_O_4_ electrode under light and dark conditions. The oxidation peak current density was significantly higher under illumination. This enhancement is attributed to the synergistic effect: under visible irradiation, photogenerated electron–hole pairs separate effectively; the holes can oxidize UA easily, while electrons transfer to the external circuit, generating a photocurrent. Fe_3_O_4_ provides a large specific surface area and abundant active sites for UA adsorption. Meanwhile, Au NPs enhance light harvesting via LSPR and facilitate electron transfer, reducing electron–hole recombination and amplifying the photocurrent signal.

### 3.3 Optimization of detection conditions for Au/Fe_3_O_4_

**FIGURE 3:**
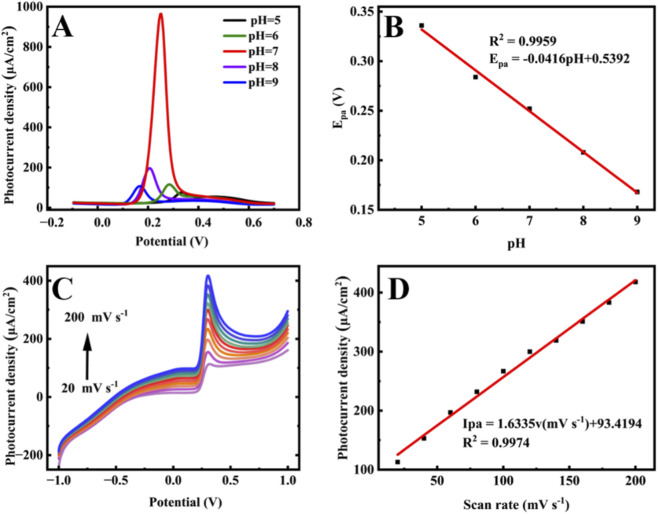
**(A)** DPVs of Au/Fe_3_O_4_ electrode in 0.1 M PBS buffer solution containing 50 μM uric acid under light conditions at different pH values (pH = 5.0, 6.0, 7.0, 8.0, 9.0), **(B)** Linear relationship between oxidation peak potential and different pH values, **(C)** Linear voltammetry curves of Au/Fe_3_O_4_ electrodes in 0.1 M PBS buffer solution (pH = 7.0) containing 50 μM urate at different scanning rates (20–200 mV∙s^−1^) under light conditions, **(D)** Linear relationship between peak oxidation current and scan rate.

The effects of pH values and scan rate on the PEC performance were investigated. Under visible light, DPV responses were recorded in PBS (pH 5.0–9.0) containing 50 μM UA (Figure 3A). The oxidation peak current increased with pH from 5.0 to 7.0, reaching a maximum at pH 7.0, and decreased at higher pH. Therefore, pH 7.0 was selected for subsequent experiments. The oxidation peak potential shifted linearly with pH changes (Figure 3B), following the equation: E_pa_ = −0.0416 pH + 0.5392 (*R* ^2^ = 0.9959). The slope is close to the Nernst value of 0.059 V/pH, indicating a one proton and one electron transfer process (Cui et al., 2017). The influence of scan rate (20–200 mV s^-1^) was studied by linear sweep voltammetry (LSV) in PBS (pH 7.0) containing 50 μM UA under illumination (Figure 3C). The oxidation peak current increased with scan rate. A linear relationship was observed between peak current (I_pa_) and scan rate (ν): I_pa_ (μA) = 1.6335ν + 93.4194 (*R* ^2^ = 0.9974) (Figure 3D), suggesting that the electron transfer is adsorption-controlled (Wang et al., 2020a).

### 3.4 Detection limit of the sensor

**FIGURE 4:**
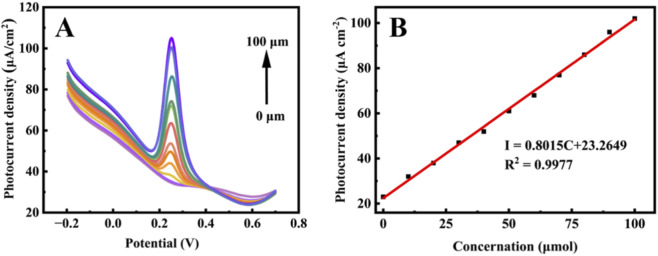
**(A)** Differential pulse voltammetry curves of Au/Fe_3_O_4_ electrode in PBS (pH = 7.0) with uric acid concentration of 0-100 μM under visible light irradiation; **(B)** Linear relationship of photocurrent intensity and concentration corresponding to Au/Fe_3_O_4_ electrode.

DPV was used to evaluate the photocurrent response at different UA concentrations (0–100 μM) in PBS (pH 7.0) under visible light (Figure 4A). The oxidation peak current increased with the increase of UA concentration. A linear calibration curve was obtained (Figure 4B): I_pa_ = 0.8015C + 23.2649 (*R* ^2^ = 0.9977), with a detection limit of 3.33 μM (S/N = 3). This demonstrates the high detection capability of the PEC sensor for UA under visible light illumination.

### 3.5 Anti-interference ability and stability of the PEC sensor

**FIGURE 5:**
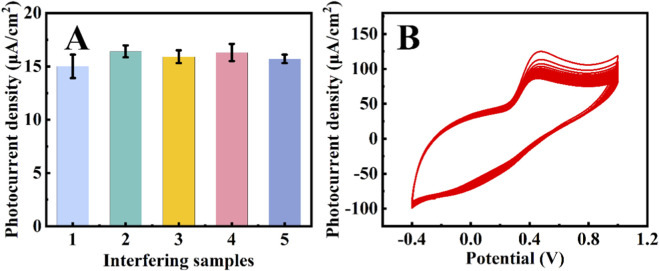
Photocurrent response of **(A)** Au/Fe_3_O_4_/GCE under visible light irradiation with 100 μM uric acid and interferers added in sequence, the concentration of interferers was 0.1 M, the sequence was as follows: 2-NaCl, 3-KCl, 4-AA, 5-Glu, and **(B)** CV curves of the Au/Fe_3_O_4_/GCE in 50 μM uric acid of 100 cycles.

To assess selectivity, potential interferent (0.1 M NaCl, KCl, ascorbic acid (AA), and glucose (Glu)) were added sequentially to PBS (pH 7.0) containing 100 μM UA (Figure 5A). The photocurrent density remained stable within an acceptable range, confirming excellent anti-interference ability and selectivity. The stability was evaluated by recording 100 consecutive CV cycles in the same solution (Figure 5B). The peak current showed only minimal degradation, indicating good stability of the Au/Fe_3_O_4_ sensor.

## 4 Conclusion

In this study, an Au/Fe_3_O_4_ nanocomposite was successfully prepared and applied in a PEC sensor for highly sensitive UA detection. Material characterization, condition optimization, and PEC tests demonstrated that the Au/Fe_3_O_4_/GCE sensor exhibits excellent PEC performance, strong anti-interference ability, and high stability, outperforming sensors based on single components. The sensor showed a linear response range of 0–100 μmol/L and a low detection limit of 3.3 μM. This performance is attributed to the synergistic effect between Fe_3_O_4_, which provides a large specific surface area, enhances visible light absorption, and improves stability, and Au NPs, which enhance light harvesting and electron transfer via LSPR, thereby amplifying the photocurrent response. The Au/Fe_3_O_4_ -based PEC sensor offers a new strategy for UA detection and holds promising application prospects in biomedical testing.

## Data Availability

The original contributions presented in the study are included in the article/supplementary material, further inquiries can be directed to the corresponding authors.
